# The mediating role of safety behavior in the relationship between safety climate and safety outcomes among sanitary workers in Pakistan

**DOI:** 10.3389/fpubh.2025.1591691

**Published:** 2025-06-11

**Authors:** Nadia Nisar, Nur Zakiah Mohd Saat, Dayana Hazwani Mohd Suadi Nata, Nurul Farahana Kamaludin, Ismarulyusda Ishak, Abdul Momin Rizwan Ahmad

**Affiliations:** ^1^Centre for Community Studies (ReaCH), Faculty of Health Sciences, Universiti Kebangsaan Malaysia, Kuala Lumpur, Malaysia; ^2^NUST School of Health Sciences, National University of Sciences & Technology (NUST), Islamabad, Pakistan; ^3^Center for Toxicology and Health Risk Studies (CORE), Faculty of Health Sciences, Universiti Kebangsaan Malaysia, Kuala Lumpur, Malaysia; ^4^Department of Health Sciences, University of York, York, United Kingdom

**Keywords:** safety climate, safety behavior, safety outcomes, multistage sampling, SmartPLS

## Abstract

**Introduction:**

Sanitary workers are regarded as the foundation of society due to their essential role in maintaining cleanliness and hygiene. In Pakistan, sanitation tasks are conducted manually, resulting in adverse health consequences for workers.

**Objective:**

This research intends to investigate the relationship between safety climate and safety outcomes among sanitary workers along with the mediating influence of safety behavior.

**Methods:**

The data was collected from two sanitation organizations in Punjab, Pakistan. The data was subsequently analyzed using SPSS version 27.0 and SmartPLS version 4. The participants were chosen from the cantonment board Attock and Lahore management company using a simple random sampling technique.

**Results/conclusion:**

The findings revealed that safety climate and its dimensions such as management commitment, safety communication learning trust, and work environment have a significant positive relationship with safety outcomes. Similarly, safety behavior significantly mediates the relationship between safety climate, dimensions and safety outcomes.

## 1 Introduction

Sanitary workers are the backbone of the sanitary system of any society (1). While performing their duties, sanitary workers faced adverse safety outcomes (2). Safety outcomes refer to all hazardous outcomes linked to sanitation activities. In developed nations, the sanitary work is predominately mechanized. However, in developing countries like India and Pakistan, sanitation work relies on human labor, particularly in urban areas with constrained resources (3).

Sanitary work-related incidents are often considered a significant issue concerning occupational health and safety (OHS). There are no global statistics about the deaths among sanitary workers (4). However, International Labor Organization (ILO) reports indicate that over 340 million workplace accidents occur globally every year (5). Turkey experienced a loss of over 12,000 people in fatal workplace accidents in the previous decade (6). In 2017, the United Kingdom and Northern Ireland recorded a total of 4,704 yearly injuries associated with sewage, waste management, and cleanup. In contrast, the United States reported 7,100 injuries in the same field during the same year (5). According to the official statistics from 2017 to late 2018, there was an average of one fatality of a sanitation worker every 5 days in India. However unfortunately, there is a lack of data about injuries specifically associated with water and sanitation in India (4).

Like other workers, sanitary workers encounter several health risks that are likely to disrupt their well-being and diminish their ability to perform their duties. Regularly visiting unhygienic locations and consistently monitoring the waste material are integral to their everyday duties. Consequently, they face various health hazards and fatalities (7). In Pakistan, there is a widespread perception that a considerable number of fatalities go unreported, particularly in remote rural regions that are far from major urban areas and get little media coverage. Numerous cases of illness among workers remain undisclosed. Authorities have not implemented a policy that would ensure the collection and preservation of information about the causes of these fatalities. Collecting and providing such information to academics and policymakers would be much valuable for establishing the specific situations that lead to sanitation workers’ death. The labor force survey conducted by the International Labor Organization revealed that in 2018, around 3,553 injuries were reported among workers in the sewage, waste management, and remediation sectors (5).

In Pakistan, occupational health safety act was approved in 2018. The aim of this act was to ensure the well-being of the workers by protecting them from health hazards by eliminating or minimizing the risks arising from the specified types of substances (8). This act has not been practically implemented in Pakistan due to the cost-cutting measures, training and lack of awareness, institutional tradition and a large workforce (9). Pakistan implemented a national sanitation plan in 2006, which is now outdated. In addition to other challenges, it failed to address the occupational health safety (OHS) challenges of the sanitation workers directly (10). Sindh, a significant commercial province in Pakistan, enacted a provincial sanitation strategy in 2015, which again does not address any of the issues about the OHS, working and living circumstances of sanitary workers in the territory.

This research incorporated three distinctive constructs including safety climate, safety behavior and safety outcomes. The safety climate refers to the perception of employees about their work environment. Safety behaviors cover the tangible actions that individuals carry out in the workplace to ensure safety. However, the first objective of this research was to investigate the relationship of safety climate and its dimensions with safety outcomes. Similarly, the second objective focused on investigating the mediating role of safety behavior in the relationship between safety climate and safety outcomes among sanitary workers in Pakistan.

Based on the above discussion this study developed the following hypothesis.

*H_1_:* There is a significant relationship between safety climate and safety outcomes.

*H_1a_:* There is a significant relationship between management commitment and safety outcomes.

*H_1b_:* There is a significant relationship between safety communication, learning trust and safety outcomes.

*H_1c_:* There is a significant relationship between work environment and safety outcomes.

In previous literature, limited studies are available on the mediating role of safety behavior in the relationship between safety climate and safety outcomes. Literature suggests that safety climate plays a crucial role in developing safety behavior among sanitary workers (2). Previous literature found a significant relationship between safety behavior and safety outcomes (11). Safety behavior also helps to mitigate adverse safety outcomes such as injuries, incidents and chronic illness among sanitary workers (1). Similarly, a previous study found that poor safety behavior also causes incidents and injuries at workplace (12). The mediating role of safety behavior among sanitary workers is a new contribution in safety climate theory. Based on the above discussion this research proposed the following hypotheses.

*H_2_:* Safety behavior significantly mediates the relationship between safety climate and safety outcomes.

*H_2a_:* Safety behavior significantly mediates the relationship between management commitment and safety outcomes.

*H_2b_:* Safety behavior significantly mediates the relationship between safety communication learning trust and safety outcomes.

*H_2c_:* Safety behavior significantly mediates the relationship between work environment and safety outcomes.

## 2 Materials and methods

### 2.1 Population and sampling

Sanitary workers from the cantonment board Taxila (CBT) and Lahore Waste Management Company (LWMC) in Punjab, Pakistan, were the population of this study. The data was collected from 187 sanitary workers in Lahore and Taxila using a simple random sampling technique. Of the 828 employees of LWMC, 152 were selected from Gulberg town, whereas, out of the 69 employees of CBT, 35 participants were selected. The selection of participants was based on an equal proportion of the data. This sample size was drawn using the Gpower software and was sufficient to represent the whole population.

### 2.2 Instrumentation and operational definitions

The research model of this study consisted of three variables. Safety climate (independent variable) covers the three dimensions; management commitment, safety communication, learning and trust, and work environment. All the safety climate dimensions were measured with five points Likert scale (Strongly Disagree to Strongly Agree). However, safety outcomes was treated as a dependent variable. Safety outcomes were measured with five points Likert scale (Never to mostly). Moreover, safety behavior was treated as a mediating variable and measured with a five-point Likert scale (Strongly Disagree to Strongly Agree).

Safety climate (SC) refers to employees’ basic perceptions in their work environment (13). In this research, SC is operationalized with three indicators such as management commitment (MC), safety communication and trust (SCT), and work environment (WE). The scale used in this research to measure SC, and its dimensions was adopted from the Nordic safety climate (14). Similarly, Safety behaviors refer to the tangible actions that individuals carry out in the workplace to ensure safety (15). In this study, the safety behavior scale was adapted from the work of He et al. (16). Moreover, safety outcomes were defined as safety activities and interventions to reduce injuries, hazards, incidents, or costs. In this research, the safety outcome scale was adapted from the work of Neal and Griffin (15). Cronbach alpha values of MC, SCLT, WE, SB and SO were 0.82, 0.70, 0.80, 0.70, and 0.77, respectively.

### 2.3 Data analysis

Data analysis was performed using SPSS version 27.0 and Smartpls version 4. Frequencies and percentages were drawn using SPSS. However, mediation analysis was performed through structural equation modeling (SEM) using SmartPLS version 4. SEM consisted of measurement and structural models. The measurement model dealt with the convergent (item loadings, AVE, CR values) and discriminant validity (HTMT) whereas the structural model dealt with hypothesis testing. The acceptable ranges of item loadings, AVE, CR and HTMT (discriminant validity) were 0.60, 0.70, and 0.90, respectively (17).

Before doing data analysis, the common method bias was checked in SPSS using Herman’s single factor analysis. The variance of the single factor was below 50% and was in the acceptable ranges (18). Similarly, according to the findings of the Mahalanobis test, this research did not find any potential outlier. The data was suitable for performing the SEM.

### 2.4 Mediation analysis

In this study, using Smart PLS, a repeated indicator approach was performed to identify and evaluate the mediating role of safety behavior (SB) in the relationship between safety climate (SC) and safety outcomes (SO). SC have three dimensions initially; it was evaluated as a second-order construct and latent variable scores of three dimensions of SC were utilized as its measurement indicators. The repeated indicator approach was employed for examining moderation because the mediation variable is a continuous variable (19).

### 2.5 Ethical considerations

The ethical approval of the study was obtained from the UKM Research Ethics Committee, Malaysia, via letter no. UKM/PPI/111/8/JEP-2022-716.

## 3 Results

Table 1 deals with the demographic profile of the participants. The findings showed that 85% of the participants were male, 60.4% of the participants belonged to the age group of 31.40 years, 75.5% of the participants had income between 25,001 to 35,000 PKR, a vast majority 81.8% of the participants were not formally educated, and 74.3% participants had experience between 6 and 10 years.

**Table 1:** Frequency distribution of the respondents with respect to their demographic profile (*N* = 187).

Demographic Profile		Frequency (F)	Percentage (%)
Gender			
	Male	159	85
	Female	28	15
Age (In Years)			
	<20 Years	20	10.7
	21–30	31	16.6
	31–40	113	60.4
	41–50	15	8
	51>	8	4.3
Income (in PKR)			
	<25,000	29	15.5
	25,001–35,000	141	75.4
	35,001>	17	9.1
Level of Education			
	Not educated	153	81.8
	Primary	23	12.3
	Matriculation	6	3.2
	Graduation	5	2.7
Experience (in years)			
	1–5	27	14.4
	6–10	139	74.3
	>10	21	11.2

### 3.1 Assessment of the measurement model

Table 2 shows the analysis of the measurement model. The findings showed that item loadings of safety climate, safety behavior and safety outcomes were in the acceptable ranges between 0.65 and 0.809. Cronbach alpha values of MC, SCLT, WE, SB and SO were 0.82, 0.70, 0.80, 0.70, and 0.77, respectively. Similarly, AVE values of MC, SCLT, WE, SB and SO were 0.53, 0.53, 0.50, 0.52, and 0.59, respectively. Moreover, CR values of MC, SCLT, WE, SB and SO were 0.82, 0.73, 0.80, 0.71, and 0.77, respectively. All the values of the measurement model were in acceptable ranges.

**Table 2:** Items loadings, alpha, average variance extracted and composite reliability (*N* = 187).

Variables	Dimensions	Variables	IL	Alpha	AVE	Cr
Safety climate (SC)						
	MC			0.82	0.52	0.82
		MC_1	0.758			
		MC_2	0.744			
		MC_3	0.756			
		MC_4	0.712			
		MC_5	0.659			
		MC_6	0.713			
	SCLT			0.70	0.53	0.73
		SCLT_1	0.548			
		SCLT_2	0.745			
		SCLT_3	0.802			
		SCLT_4	0.793			
	WE			0.80	0.50	0.80
		WE_1	0.673			
		WE_2	0.744			
		WE_3	0.700			
		WE_4	0.774			
		WE_5	0.669			
		WE_6	0.678			
Safety Behavior (SB)				0.70	0.52	0.71
		SB_1	0.757			
		SB_2	0.735			
		SB_3	0.709			
		SB_4	0.698			
Safety outcome (SO)				0.77	0.59	0.77
		SO_1	0.764			
		SO_2	0.772			
		SO_3	0.809			
		SO_4	0.743			

IL, items loadings; AVE, average variance extracted; CR, composite; MC, management commitment; SCLT, safety communication, learning and trust; WE, work environment; SB, safety behavior; SO, safety outcomes.

Table 3 shows the discriminant validity of the latent variables. The findings showed that all the values of the latent variables are below 0.90 and are in the acceptable ranges.

**Table 3:** Discriminant validity of the latent constructs through HTMT ratio (*N* = 187).

Constructs	MC	SCLT	WE	SB	SO
MC	–				
SCLT	0.539	–			
WE	0.423	0.492	–		
SB	0.737	0.648	0.533	–	
SO	0.50	0.70	0.55	0.657	–

### 3.2 Second-order construct establishment

This research consists of three latent variables such as safety climate (SC), safety behavior (SB), and safety outcome (SO). It is important to note that SC is a higher-order reflective-formative construct. Higher-order models commonly incorporate testing second-order structures which comprise two layers of components (17). In this research, SC have three sub-dimensions such as Management commitment (MC), Safety communication, learning and trust in co-workers’ safety competence (SCLT), and work environment (WE).

Three steps are involved in assessing formative measures: (1) test for weight significance (*t*-value), (2) test for multi-collinearity (VIF) and (3) Indicator Weights (19, 20). Table 4 presents the findings relating to the formation of safety climate (SC) as the second-order construct in this study. The findings show no indication of collinearity among the indicators. All the values are less than the threshold of VIF = <5.0. All the dimensions of the second-order construct were retained because all the dimensions are important to operationalized the variables, and it was in accordance with the composite method of Bollen and Bauldry (21).

**Table 4:** Second order second-order construct establishment (*N* = 187).

Second order	Dimensions	VIF	Beta	*t*-value	*P*-value
SC	MC	1.68	0.476	5.481	0.000
	SCLT	1.31	0.467	4.650	0.000
	WE	1.11	0.344	4.275	0.000

### 3.3 Assessment of the structural model

After establishing the outer model, the next step was to assess the inner model which involves testing hypotheses by calculating path coefficients and t-values. Using Smart PLS 4, the current study applied a bootstrapping process with 5,000 resamples to ascertain the significance of path coefficients to test hypothesized relationships as indicated by Chin et al. (22). WHO suggested that 200 to 1,000 bootstrap samples result in sufficient standard error estimates. To determine the significance of the path coefficient, the present study depended on the bootstrapping method which was set in Smart PLS software. The estimates of the complete structural model can be seen in Supplementary Figure 1. A *p*-value below 0.05 indicated acceptance of the hypothesis, while a *p*-value above 0.05 indicated rejection of the hypothesis.

### 3.4 Hypothesis testing

H1 was established to check the association between safety climate (SC) and safety outcomes (SO). The results in Table 5 and Supplementary Figure 1 confirm a significant positive relationship between SC and SO (*β* = 0.622, *t* = 10.616, *p* < 0.01). Based on the results, H1 is accepted.

**Table 5:** The relationship between safety climate, safety outcomes and the mediating role of safety behavior (*N* = 187).

Hypo	The path	Beta value	*t*-value	*p*-value	Decision	*R* ^2^	*Q* ^2^
H_1_	SC→SO	0.622	10.616	0.000	Supported	0.387	0.210
H_1a_	MC → SO	0.171	2.068	0.039	Supported	0.374	
H_1b_	SCLT→SO	0.363	3.490	0.000	Supported		
H_1c_	WE→SO	0.254	3.238	0.000	Supported		

Hypo	The path	*t*-value	*p*-value	Mediation	Decision	*R* ^2^	*Q* ^2^
H_2_	SC→SB→SO	2.086	0.037	Supported	Accepted	0.389	0.216
H_2a_	MC→SB→SO	2.278	0.010	Supported	Accepted	0.040	
H_2b_	SCLT→SB→SO	2.218	0.029	Supported	Accepted		
H_2c_	WE→SB→SO	2.001	0.045	Supported	Accepted		

The relationship between safety climate (SC) management commitments (MC), Safety communication, learning and trust (SCLT), work environment (WE), safety behavior and safety outcomes (SO).

Table 5 and Supplementary Figure 2 show the first-order direct hypotheses. In first-order direct hypotheses, various dimensions of safety climate (SC) were tested with safety outcomes (SO). H_1a_ confirmed a significant positive relationship between MC and SO (*β* = 0.0171, *t* = 2.068, *p* = 0.039). H_1b_ confirmed a significant positive relationship between SCLT and SO (*β* = 0.363, *t* = 3.490, *p* = 0.000). Similarly, H_1c_ also confirmed a significant positive relationship between WE and SO (*β* = 0.254, *t* = 3.238, *p* < 0.001). Based on the results, H_1a_, H_1b_ and H_1c_ were failed to be rejected.

### 3.5 Mediation analysis

In Table 5, and Supplementary Figure 3 the findings of H^2^ confirmed that safety behavior significantly mediated the relationship of safety climate and safety outcomes (*t* = 2.086, *p* = 0.037). H_2a_ results revealed that safety behavior significantly mediated the relationship between management commitment and safety outcomes (*t* = 2.278, *p* = 0.010). Similarly, the findings of H_2b_ revealed that SB significantly mediates the relationship between SCLT and SO (*t* = 2.218, *p* = 0.029). Moreover, in H_2c_, SB significantly mediated the relationship between WE and SO (*t* = 2.001, *p* = 0.045). Based on the results, H_2_, H_2a_, H_2b_, and H_2c_ were failed to be rejected with mediating effects.

## 4 Discussion

This study was conducted to explore the mediating role of safety behavior in the relationship between safety climate and safety outcome among sanitary workers. The study was conducted in two cities in Punjab Pakistan. A total of eight hypotheses were developed to investigate the documented issue.

H_1_ was developed to determine the association between safety climate and safety outcome. The findings revealed that there is a significant positive relationship between both attributes. This finding is consistent with prior literature. For instance, numerous studies demonstrated that a positive safety climate is associated with positive safety outcomes (13, 23), because a safety climate reduces negative workplace outcomes such as incidents, injuries, and increases compliance with safety protocols (11). When employees perceive a strong commitment to safety from their organization and believe that safety is a top priority, they are more likely to adopt safe work practices, report safety concerns, and actively participate in safety-related activities (24). This, in turn, reduces the likelihood of accidents and injuries, as employees are more vigilant and proactive in identifying and mitigating potential safety hazards (25). Furthermore, a positive safety climate can foster a sense of psychological safety, where employees feel comfortable speaking up about safety issues without fear of repercussions, which can further enhance the organization’s ability to identify and address safety-related concerns (11).

Management commitment (MC) is an important dimension of the safety climate that influences safety outcomes (26). Management commitment refers to employees’ opinions of how much their managers appreciate and support safe working conditions and are committed to their safety and well-being (13). Our study formulated a hypothesis (H_1a_) to check the relationship between management commitment and safety outcomes. The findings showed a positive association between management commitment and safety outcomes. The findings are in line with the previous literature. For example, research revealed that management commitment reduces workplace injuries by promoting safety behaviors (27). Similarly, another research study depicted that MC promotes teamwork, safety improvements, feedback, and learning that fosters the workers’ safety behavior and reduces the adverse effects of safety outcomes (28). Furthermore, literature has demonstrated that management commitment helps sanitary personnel avoid hazardous safety outcomes by ensuring and implementing safety policies (29).

Hypothesis H_1b_ was constructed to verify the relationship between safety communication and safety outcomes. The findings confirmed a significant positive relationship between both attributes. These findings are also consistent with the previous literature. For example, a study revealed that a safety communication strategy forewarns potential risks, and unsafe work practices and offers helpful recommendations to foster change in the workplace to avoid occupational hazards (30). A study from Pakistan depicted that effective communication is crucial for establishing a secure and favorable work environment. Utilizing advanced technologies like mobile emergency and social network response systems may enhance communication and promote safety outcomes by reducing occupational hazards (31). Similarly, safety communication before starting work can empower workers, reduce workplace injuries, and significantly improve safety outcomes (32). Safety communication allows workers to inquire about and resolve any uncertainties they may have on security measures (33). Moreover, safety communication is important because it promotes a safety culture, which helps reduce the number of workplace incidents and boosts the employees’ well-being (34).

H_1c_ was developed to investigate the relationship between work environment and safety outcomes. The findings confirmed a significant positive relationship between both attributes. These findings are consistent with the previous literature; for instance, an earlier study revealed that individuals who work in high-risk environments face adverse health outcomes such as incidents or chronic illness. These individuals also learn from their working experiences to avoid future occupational incidents (35). Another previous study found that the working environment predicts adverse health outcomes among sanitary workers in various regions of the world, including Asia and Pakistan, such as hepatitis, lung infection, and psychological disorders (36). Similarly, research conducted in Pakistan demonstrated that sanitary workers often face abusive language from managers and higher authorities, which is ultimately associated with adverse psychological outcomes such as cognitive failure (37). A recent study conducted in India concluded that due to working conditions, sanitary workers are facing various psychological impacts, such as depression and stress (38).

Hypothesis H_2_ was formulated to determine the mediating role of safety behavior in the relationship between safety climate and safety outcomes. The findings indicate that safety behavior mediates the relationship between safety climate and safety outcomes. The findings of this study further support the research conducted by Lyu et al. (11), which concluded that safety climate is a significant predictor of safety behavior. The relationship between safety climate and work accidents is influenced by safety behavior. However, some other studies demonstrated that safety climate directly influences safety behavior, reducing negative safety outcomes such as injuries and workplace incidents (39). Similarly, safety climate has a significant impact on workers’ behavior and plays a crucial role in minimizing accidents (11). Moreover, another study revealed that safety climate influences workers’ safety behavior, reducing occupational incidents (40).

Hypothesis H_2a_ was formulated to investigate the mediating role of safety behavior in the relationship between management commitment and safety outcomes. The findings indicate that safety behavior mediates the relationship between management commitment and safety outcomes. The findings of H_2a_ is a new contribution to existing literature because previous studies did not justify this relationship. However, management commitment frequently entails the establishment of well-defined safety policies and procedures. When employees perceive resilient management support for these policies, they tend to be more inclined to adhere to safety rules and regulations. Adhering to regulations and guidelines can significantly decrease the chances of accidents and injuries, resulting in improved safety outcomes (39). Similarly, Management commitment plays a crucial role in fostering a safety culture where workers are actively engaged in safety behavior. This involves reporting potential hazards, active participation in safety training sessions, and active involvement in safety committees. Engaging in a safe environment improves safety outcomes (41).

Safety communication is essential to develop trust among the sanitary worker. Safety communication helps the sanitary worker to reduce workplace injuries. In this lieu, the H_2b_ was formulated to investigate the mediating role of safety behavior in the relationship between safety communication and safety outcomes. The findings indicate that safety behavior mediates the relationship between safety communication and safety outcomes. H_2b_ is a new contribution in theory because previous literature did not support this relationship. Based on the findings of H_2b_, it is suggested that the findings be repeated to get fruitful results. The mediating role of safety behavior is important due to the following reasons, Firstly, it enhances the opportunities of effective communication and helps to opt the safety measures for positive safety outcomes. Secondly, safety behaviors, such as following safety procedures, reporting hazards and using personal protective equipment (PPE), directly influence the possibility of injuries and accidents. Conversely, ineffective safety communication promotes unsafe behaviors such as ignoring safety rules and shortcutting procedures, which increase the risk of adverse safety outcomes.

Hypothesis H_2c_ was developed to determine the mediating role of safety behavior in the relationship between work environment and safety outcomes. The findings indicate that safety behavior mediates the relationship between work environment and safety outcomes. Similar to the findings of H_2c_, a previous study revealed that consistent reinforcement of feedback and communication from managers in a supportive safety environment develops safety behavior among sanitary workers. When workers adhere to safety rules and participate in safety activities, they are more inclined to persist in these behaviors, resulting in long-term gains in safety outcomes (42). Similarly, a previous study reported that a supportive safety environment supports safe behavior, leading to improved safety results and mitigating safety hazards (39). Similarly, the literature also demonstrated that the safety environment influence on safe safety outcomes is mediated by participation and compliance (43).

### 4.1 Limitations

This research has the following limitations. First, this study is limited to sanitary workers who work in public sector organizations. In the future, the scope of the project can be enhanced by involving sanitary workers in private organizations. Similarly, a comparison can be made between both organizations. Secondly, this study focused on a single province, Punjab, Pakistan. However, similar studies should be conducted in other provinces of Pakistan, such as Sindh, Balochistan, Khyber Pakhtunkhwa, and Gilgit Baltistan. Comparative analysis of various provinces regarding safety climate, safety behavior, and safety outcomes will become more beneficial to improving sanitary work in Pakistan. The sample size of this research is limited to 187 participants. In future research, this sample size can be enhanced. Similarly, longitudinal research can also be inferred to explore this problem more ethically. Thirdly, the data may have self-reported biases as there was no way to verify the responses. Finally, this study does not imply any causality because it was a cross-sectional research design.

### 4.2 Strengths

Our study involved sanitary workers in Pakistan, which is an extremely important, but unfortunately, highly neglected population group. To our best knowledge, this was one of the first studies in this domain, that was conducted in the Punjab province. Our research incorporated three distinctive constructs of safety performance of sanitary workers including safety climate, safety behavior and safety outcomes.

## 5 Conclusion

Our research provides robust evidence that the safety climate exerts a significant positive influence on safety outcomes. Notably, the analysis reveals that safety behavior functions as a mediating variable, strengthening the link between safety climate (and its constituent dimensions) and safety outcomes among sanitary workers. This mediation suggests that improvements in the safety climate are likely to enhance safety behaviors, which in turn may lead to better safety outcomes. These findings carry important implications for policymakers and organizational leaders, highlighting the necessity of targeted interventions that address both the structural and behavioral aspects of workplace safety especially keeping in view the sanitary workers. By focusing on enhancing management commitment, fostering open safety communication, building trust, and improving the work environment, organizations can more effectively shape the safety behaviors of sanitary workers. Consequently, this research underscores the critical role of comprehensive safety policies and procedures in not only shaping intentions but also in achieving tangible improvements in occupational safety for this workforce.

## Data Availability

The raw data supporting the conclusions of this article will be made available by the authors, without undue reservation.
