# Supplementary material for: The mediating role of safety behavior in the relationship between safety climate and safety outcomes among sanitary workers in Pakistan
Source: Front Public Health. 2025 Jun 11;13:1591691. doi: 10.3389/fpubh.2025.1591691 (PMC12187649; doi:10.3389/fpubh.2025.1591691)
Supplement: Supplementary file 1 [file Data_Sheet_1.docx]

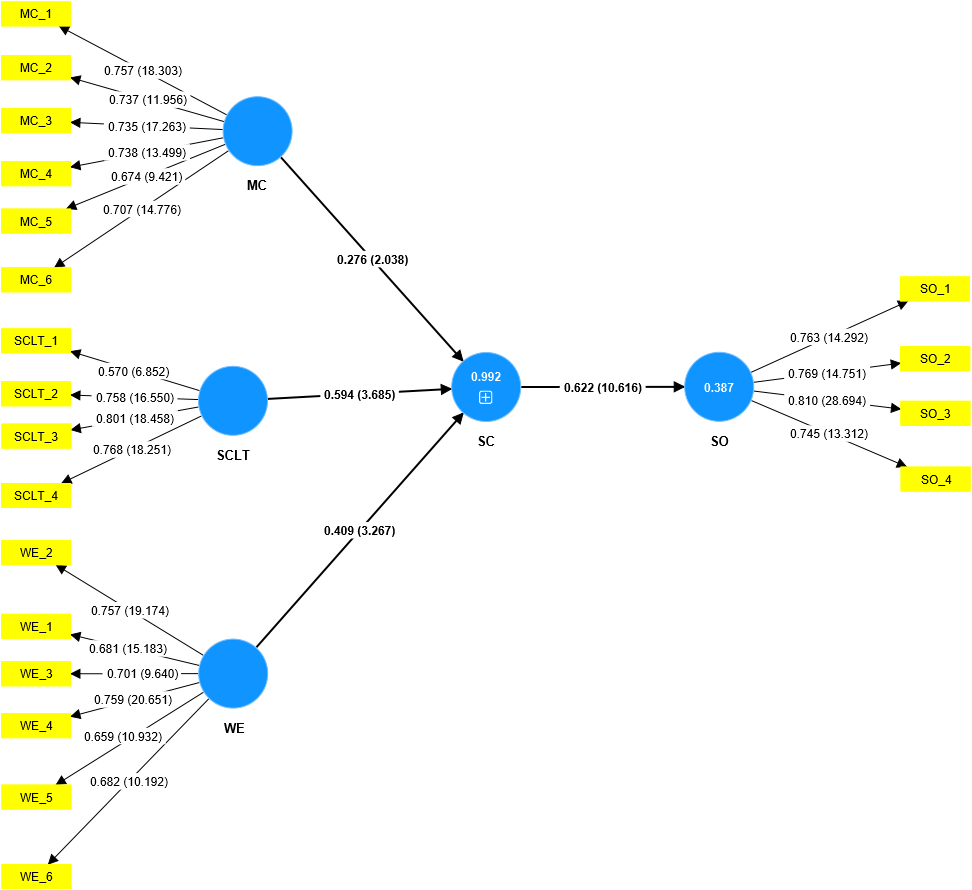


**^Supplemental Figure 1: Relationship between safety climate (SC) and safety outcomes (SO). MC refers to management commitment, SCLT refers to safety communication and trust, and WE refer to the working environment. However, MC, SCLT, and WE are the dimensions of safety climate (SC).^**


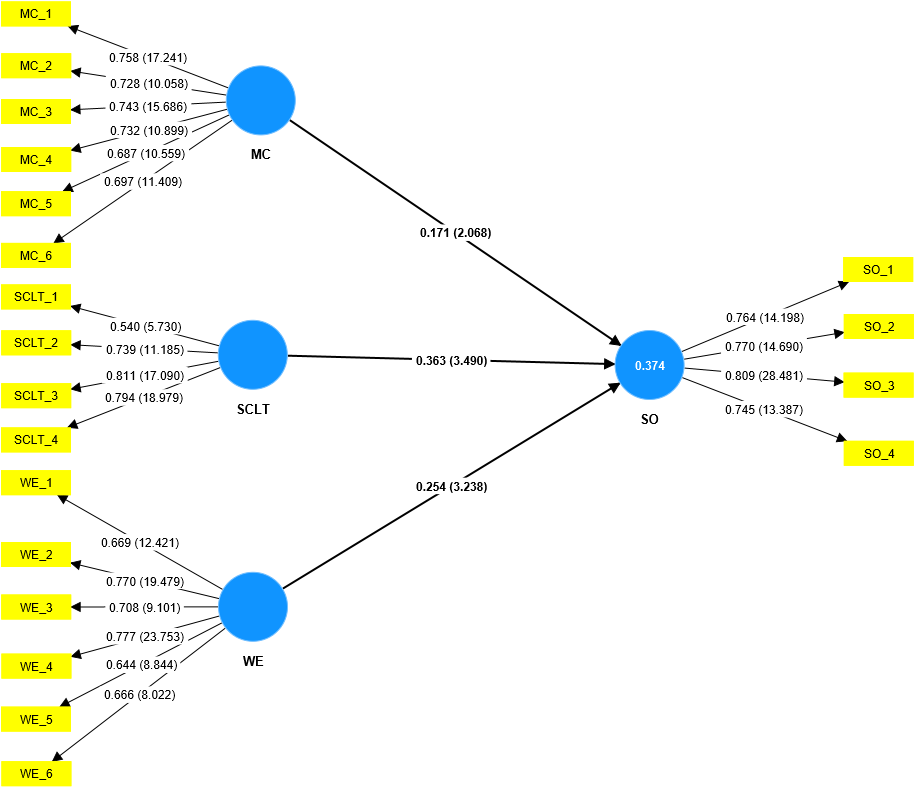


**^Supplemental Figure 2: Relationship between various dimensions of safety climate (SC) and safety outcomes (SO). MC refers to management commitment, SCLT refers to safety communication and trust, and WE refer to the working environment. However, MC, SCLT, and WE are the dimensions of safety climate.^**

**
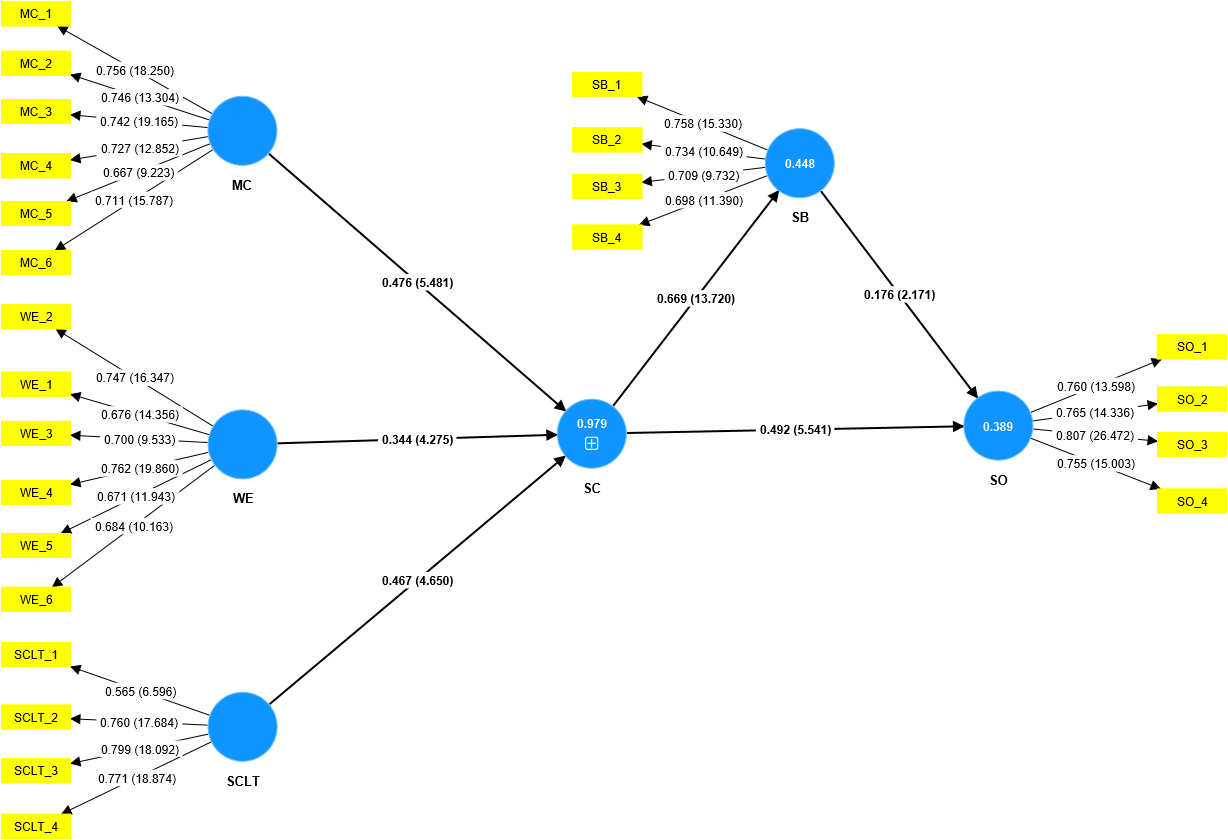
**

**^Supplemental Figure 3: The mediating effect of safety behavior (SB) in the relationship between safety climate (SC ) and safety outcome (SO). MC refers to the management commitment, SCLT refers to the safety communication and trust, and WE refer to the working environment. However, MC, SCLT, and WE are the dimensions of safety climate (SC).^**
